# Ochronotic arthropathy effectively treated with total hip and total knee arthroplasty: a case report

**DOI:** 10.3389/fmed.2023.1212580

**Published:** 2023-09-19

**Authors:** Yikai Liu, Chenkai Li, Zian Zhang, Xinzhe Lu, Haining Zhang

**Affiliations:** ^1^Department of Joint Surgery, The Affiliated Hospital of Qingdao University, Qingdao, China; ^2^Department of Orthopedics, Peking Union Medical College, Beijing, China

**Keywords:** ochronotic arthropathy, total hip arthroplasty, alkaptonuria, total knee arthroplasty, joint pain

## Abstract

Ochronosis is a rare autosomal recessive disorder of tyrosine metabolism characterized by multilevel spinal degeneration and arthritis of large weight-bearing joints, which is referred to as ochronotic arthropathy. In this case report, we describe diagnosis and treatment of ochronotic arthropathy in a patient who underwent total hip arthroplasty (THA) and total knee arthroplasty (TKA). The Harris hip score was 26 preoperatively and 45, 68, 76, 90, 92, and 94 at 1, 3, 6, 9, 11, and 14 months, respectively, postoperatively. The forgotten joint score (FJS) of the hip was 27.8, 52.8, 81.1, 89.0, 90.6, and 92.4 at 1, 3, 6, 9, 11, and 14 months, respectively, postoperatively. TKA was performed 8 months after THA. The Knee Society Score was 36 before TKA and 74, 82, and 90 at 1, 3, and 6 months, respectively, after TKA. The FJS of the knee was 36.6, 63.9, and 84.5 at 1, 3, and 6 months, respectively, after TKA. The patient’s knee range of motion returned to normal, with significant reduction in pain and improved satisfaction levels after TKA. THA and TKA can achieve good clinical outcomes in patients with ochronosis accompanied by severe joint pain.

## Introduction

Ochronosis (also referred to as alkaptonuria) is a term coined by Virchow who first reported this condition in 1866 based on autopsy studies. It is a rare autosomal recessive hereditary disease with an incidence of only 1:125,000–1:1 million patients worldwide. Ochronosis is characterized by deposition of large amounts of homogentisic acid (HGA, an intermediary product of tyrosine and phenylalanine metabolism) in connective tissues such as skin, cartilage, ligaments, and even cardiac valves, as well as urolithiasis, which results in dark-colored urine (1). An acidic environment produces an inflammatory response in joints and disturbs articular cartilage metabolism, which promotes cartilage degeneration. The thoracolumbar spine is usually affected first, followed by the peripheral joints, typically the knee, hip, and shoulder joints. The main clinical symptoms include pain, stiffness, and disability in the affected areas (2).

## Case description

A 60-year-old man with a 10-month history of severe right hip pain and a 50-year history of allergic asthma presented with slight low back discomfort over 1 month and unknown duration of glucocorticoid use. Physical examination did not reveal dark pigmentation of the sclera or bluish-brown discolouration of the auricles. The “4” character test was performed through the following procedures: the patient was told to lie flat on their back, extend one leg and lift the other leg onto the knee of the extended leg. Then we let the patient bend and press down the knee (the legs form a “4” character) to observe whether it can induce pain in the hip on the same side. The “4” character test showed bilaterally positive results, which indicated that the disease had affected hip and sacroiliac joint. The Bragard’ s test was negative, which indicated that there is no compression of the sciatic nerve.

**FIGURE 1:**
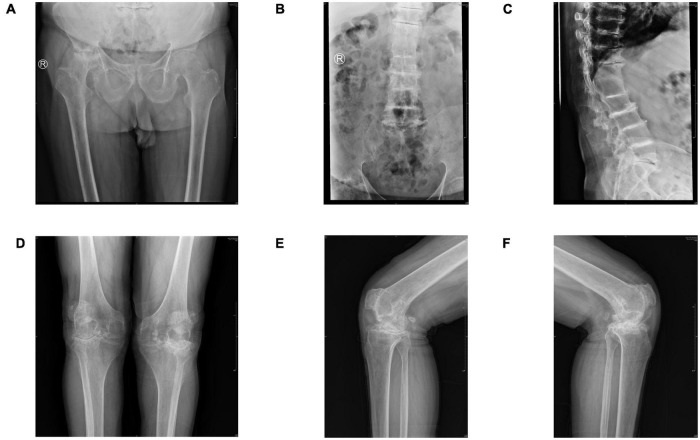
**(A)** Pelvic radiograph (anteroposterior view) showing an excessive femoral neck-stem angle and fusion of the right femoral head and pelvis. Extensive hyperosteogeny of the right femoral head is observed. **(B,C)** Lumbar radiographs (anteroposterior and lateral views) showing multilevel narrowing of the thoracic and lumbar intervertebral spaces and lumbar spondylolisthesis, with osteophytosis, calcification, and subchondral osteosclerosis. **(D)** Radiograph of the knee (anteroposterior view) showing severe degeneration, extensive osteophytosis, significant joint space narrowing, and subcortical osteosclerosis. **(E,F)** Preoperative radiographs of the bilateral knees (lateral views) showing narrowed joint spaces, loose bodies, severe subchondral osteosclerosis, and extensive osteophytosis.

Imaging revealed an excessive femoral neck-stem angle, right hip ankylosis, and extensive hyperosteogeny of the right femoral head (Figure 1A). Spinal radiography showed multilevel narrowing of the thoracic and lumbar intervertebral spaces and lumbar spondylolisthesis with osteophytosis, calcification, and subchondral osteosclerosis (Figures 1B, C). Standing radiographs of the bilateral knees showed narrowing of the femoral-patellar space laterally and of the femoral-tibial compartment in the anterior-posterior position; notably, the joint space was completely narrowed in the left knee and medial femoral-tibial compartment of the right knee. We observed extensive osteophytosis of the distal femur, tibial plateau, and patella, and a loose body was identified in the posterior articular capsule. We detected no radiographic abnormalities of the sacroiliac joints or osteoporosis (Figures 1D–F).

**FIGURE 2:**
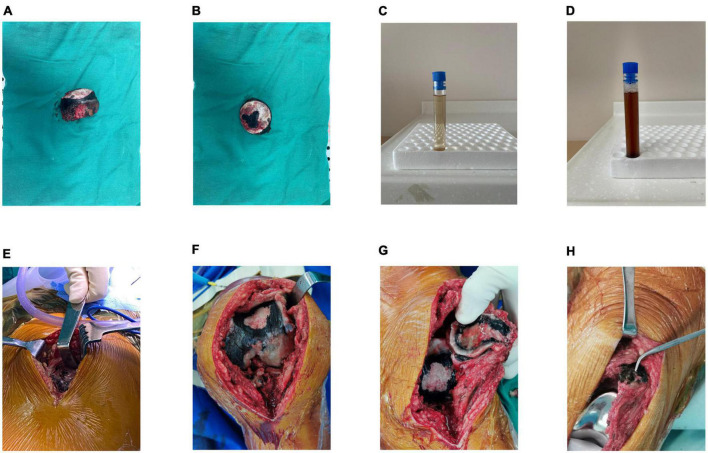
**(A,B)** Intraoperative images showing severe destruction of the femoral head cartilage and darkened subchondral bone. **(C)** Naked-eye examination of urine showing normal appearance of the urine immediately after sample collection. **(D)** Image obtained after exposure of urine to air for 24 h showing darkening of urine color. **(E)** Intraoperative images showing darkening of the soft tissue to a brown and black color. **(F)** Image showing pigmentation on the articular surface of the femur. **(G)** Image showing pigmentation on the articular surface of the patella. **(H)** Image obtained after prosthesis placement.

The color of fresh urine did not differ from that of urine obtained from healthy individuals; however, urine samples that were allowed to stand appeared chocolate-brown 24 h later (Figures 2C, D). Blood and urine tests did not reveal any abnormalities in biochemical indices.

We performed total hip arthroplasty (THA) under general anesthesia using a direct lateral approach. The operation time was 130 min, and blood loss was approximately 200 mL. Intraoperatively, the subcutaneous tissue and tensor fascia lata appeared normal in color. Removal of superficial tissues revealed dark spots on the gluteus medius, gluteus minimus, and the hip joint capsule appeared brown (Figure 2E). The remaining cartilage in the non-weight-bearing area of the resected femoral head appeared dark black. In the weight-bearing area, the cartilage was worn out and rough and appeared black, and normal-colored subchondral bone was exposed (Figures 2A, B). Therefore, macroscopic diagnosis of ochronosis was made.

**FIGURE 3:**
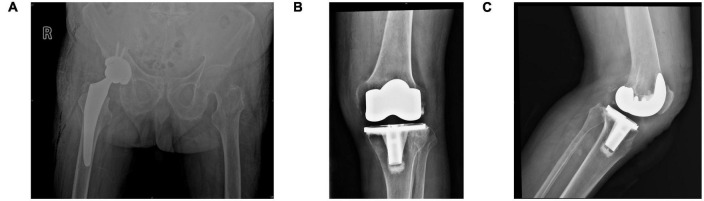
**(A)** Bedside hip radiograph (anteroposterior view) obtained the day following surgery showing anteversion and abduction angles within the normal range based on the Lewinnek and Callanan safe zones. **(B,C)** Radiographs of the knee (anteroposterior and lateral views) obtained the day following TKA showing correct prosthesis placement. TKA, total knee arthroplasty.

Intravenous antibiotics were administered 3 days postoperatively. A drainage tube was inserted, clipped for 2 h, and drained for 24 h postoperatively. Partial weight bearing using crutches was introduced on the second postoperative day, and the patient progressed to full weight bearing after 1 week. Plain pelvic radiographs showed correct positioning and size of the prosthesis, with accurate measurements of the anteversion and abduction angles (Figure 3A).

Owing to severe knee pain, we performed left total knee arthroplasty (TKA) under general anesthesia combined with an intraoperative nerve block using the medial parapatellar approach, 8 months after THA. Similar to intraoperative findings during THA, we observed cartilage and synovial pigmentation (Figures 2F–H). Balanced extension and flexion gaps were obtained after prosthesis placement. The knee range of motion was 0–140°. A drainage tube was inserted, clipped for 2 h, and retained over 24 h postoperatively. Out-of-bed activity was permitted on postoperative day two. Plain radiographs of the knee obtained the day following surgery showed correct positioning of the prosthesis (Figures 3B, C). The patient’s pain was significantly relieved 1 month postoperatively, accompanied by remarkable patient satisfaction.

**FIGURE 4:**
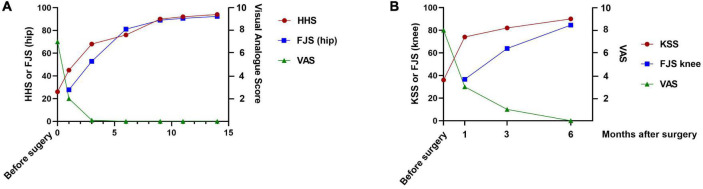
**(A)** Graph showing the HHS, FJS (hip) and VAS scores obtained preoperatively and at the 1-, 3-, 6-, 9-, 11-, and 14-month follow-ups. **(B)** Graph showing the KSS, FJS (knee), and VAS scores obtained preoperatively and at the 1-, 3-, and 6-month follow-ups. FJS, forgotten joint score; HHS, Harris hip score; KSS, Knee Society Score; VAS, visual analog score.

We used the visual analog scale (VAS), Harris hip score (HHS), and forgotten joint score of the hip (FJS hip) for follow-up evaluation at 1, 3, 6, 9, 11, and 14 months after THA, based on the recommendations of Nilsdotter et al. (3). The VAS score was 7 preoperatively, 2 at 1 month, and 0 at subsequent follow-ups. The HHS score was 26 preoperatively and 45, 68, 76, 90, 92, and 94 at 1, 3, 6, 9, 11, and 14 months, respectively, after the THA. The FJS (hip) was 27.8, 52.8, 81.1, 89.0, 90.6, and 92.4 at 1, 3, 6, 9, 11, and 14 months, respectively, after the THA (Figure 4A).

We evaluated the VAS, Knee Society Score (KSS) and the FJS (knee) 1, 3, and 6 months after TKA. The VAS score was 8 preoperatively, 3 at the 1-month, 1 at the 3-month, and 0 at the 6-month follow-up. The KSS was 36 preoperatively, 74 at the 1-month, 82 at the 3-month, and 90 at the 6-month follow-up, respectively. The FJS (knee) was 36.6, 63.9, and 84.5 at the 1-, 3- and 6-month follow-ups, respectively (Figure 4B).

## Discussion

This case report (which included short-term follow-up) highlights the effectiveness of THA and TKA for management of ochronotic arthropathy. The HHS and FJS (hip) scores were significantly lower at the 6-month follow-up. Notably, evaluation of the HHS after THA but before TKA showed that the affected leg and the functional scores did not improve significantly, which may be attributed to progressive knee arthritis. Bilateral knee pain was fairly severe and affected the patient’s sleep and restricted walking and affected the patient’s quality of life. TKA was followed by remarkable improvement in the HHS.

Chronic joint pain may develop in individuals between 30 and 40 years of age; the mean age at joint arthroplasty was 53 years in patients with ochronosis and 67 years in those with osteoarthritis (OA), (4) which indicates that this metabolic disorder is associated with accelerated cartilage degeneration. The severity of radiological manifestations does not correspond with the clinical symptoms during early-stage ochronotic arthropathy (5). Despite severe multilevel narrowing of the intervertebral spaces and intervertebral disk calcification, our patient did not present with clear evidence of low back pain. Some patients with chronic low back pain may be misdiagnosed with ankylosing spondylitis owing to the similar clinical and radiographic presentation (6).

Alkaptonuria must be considered in the differential diagnosis in patients with narrowing of > one intervertebral space or obvious degenerative changes observed during the fifth and sixth decades of life, even in clinically asymptomatic cases. Ochronosis involving the spine can be distinguished from ankylosing spondylitis based on sacroiliac joint evaluation on spinal radiography; the spine usually remains unaffected in the former condition. The possibility of OA should be considered in patients with short-term joint space changes and rapid progression of joint dysfunction.

The diagnosis of this disease mainly depends on macroscopic changes, and ochronotic arthropathy can be confirmed based on quantitative measurement of HGA in the urine and mutation analysis of the *HGD* gene. Whether it is essential to do this mutation analysis remains to be discussed because it is not economic while hardly yield any change for the treatment. Arthroscopy can effectively diagnose and treat suspected cases of ochronotic arthropathy. Arthroscopic diagnosis and treatment can alleviate joint pain and improve range of motion (7).

The mechanisms underlying cartilage destruction and progressive OA remain largely unknown in this condition characterized by metabolic dysfunction. Oxidation of HGA results in formation of benzoquinone acetic acid, which inhibits lysine hydroxylase, thereby reducing the cross-linkage between collagen fibers. Deeper understanding of the pathogenetic contribution of this pathway to the development of ochronotic arthropathy may facilitate administration of specific drugs to prevent lysine hydroxylase inhibition and therefore treat ochronotic arthropathy (5). Ligament destruction reduces joint stability, which further aggravates cartilage abrasion. Currently, drugs for management of progressive ochronotic arthropathy remain unavailable. Nitisinone (Orfadin), which inhibits the 4-hydroxyphenylpyruvic acid dioxygenase enzyme (an important component of the tyrosine catabolic pathway) is a promising drug for treatment of ochronosis and was approved by the Food and Drug Administration in 2002 for the treatment of hereditary tyrosinemia. Nitisinone treatment reduced plasma HGA levels and prevented ochronosis in mouse models (8) and in patients with alkaptonuria (9). However, whether nitisinone therapy may reverse ochronotic arthropathy remains unclear. Vitamin C intake (up to 1 g/day) can suppress urinary excretion of benzoquinone acetic acid; however, long-term efficacy of this approach remains unproven. Restriction of tyrosine and phenylalanine intake can reduce HGA excretion but increases the risk of nutritional deficiencies.

Short-term follow-up showed good outcomes of THA and TKA in our patient with ochronotic arthropathy accompanied by severe hip pain. However, most patients show pathological changes during surgery, and the condition is diagnosed only through continued follow-up of the patient’s history supported by auxiliary evaluation (10). Currently, an effective diagnostic method remains unavailable for early detection of ochronotic arthropathy. Our case report emphasizes that clinicians should be familiar with the pathological mechanism underlying ochronotic arthropathy, the possible outcomes, and importance of accurate detailed history taking with regard to the patient’s personal and family histories.

There are a few international studies reporting hip (11), knee (12), shoulder (13) arthroplasty for ochronotic arthropathy. However, the overall number of cases remains small and further accumulation of cases, extensive discussions, and long-term follow-up are warranted in clinical practice. This case report provides valuable information regarding the effects of ochronotic disease on cartilage and surrounding tissues, and our findings may improve the daily clinical practice and management of ochronotic patients for the comprehensive diagnosis and treatment of ochronotic arthropathy.

## Data availability statement

The original contributions presented in this study are included in the article/supplementary material, further inquiries can be directed to the corresponding author.

## Ethics statement

The studies involving humans were approved by the Medical Ethics Committee of the Affiliated Hospital of Qingdao University. The studies were conducted in accordance with the local legislation and institutional requirements. The participants provided their written informed consent to participate in this study. Written informed consent was obtained from the individual(s) for the publication of any potentially identifiable images or data included in this article.

## Author contributions

YL: Conceptualization, Writing—review & editing, Writing—original draft. CL: Methodology, Writing—review & editing. ZZ: Formal analysis, Investigation. XL: Formal analysis, Investigation. HZ: Funding acquisition, Resources, Supervision.
